# *Onthophagus cervicornis* Kirby, 1825, new synonym under *Onthophagus dama* (Fabricius, 1798) (Coleoptera, Scarabaeidae, Scarabaeinae)

**DOI:** 10.3897/zookeys.419.7849

**Published:** 2014-06-24

**Authors:** Michele Rossini, Fernando Z. Vaz-de-Mello, Darren J. Mann

**Affiliations:** 1 Università degli Studi di Urbino Carlo Bo, Dipartimento di Scienze della Terra, della Vita e dell'Ambiente (DiSTeVA), Campus Scientifico Enrico Mattei, via Ca' Le Suore 2/4, 61029, Urbino (PU), Italy; 2 Muséum National d’Histoire Naturelle, Département Systématique et Évolution, Entomologie, Paris, France. Permanent Address: Universidade Federal de Mato Grosso, Instituto de Biociências, Departamento de Biologia e Zoologia, Av. Fernando Corrêa da Costa, 2367, Boa Esperança, 78060-900 - Cuiabá, MT, Brazil; 3 Hope Entomological Collections, Oxford University Museum of Natural History, U.K.

**Keywords:** Dung beetles, North America, India

## Abstract

After examining syntypes of *Onthophagus cervicornis* Kirby, 1825, previously considered to be a synonym of the North American *Onthophagus striatulus* (Palisot de Beauvois, 1809), we confirm the true identity and new synonymy under South Asian *Onthophagus dama* (Fabricius, 1798).

## Introduction

Kirby (1825: 565) described *Onthophagus cervicornis* in a paper of new species and genera that were soon to be published in the “introduction to entomology” (Kirby 1826). The specimens of *Onthophagus cervicornis* were cited by Kirby (1825: 565) as originating from “Ex. Mus. D. Francillon”. The collection of John Francillon (1744–1818) was sold at auction by King in 1816 (duplicate insects, 253 lots) and 1818 (Foreign Insects, 1328 lots) (Chalmers-Hunt 1976: 76–77) and it must have been at this second sale, after the death of Francillon that Kirby, who was known to frequent these auctions, purchased the specimen(s). Kirby (1826: 310) stated “I have a beautiful little specimen in my cabinet, (I believe collected by Mr. Abbott of Georgia,) in which the horns have a lateral tooth, or short branch, like those of a stag; and which I have therefore named O. cervicornis.” The discrepancy between Kirby (1825: 565) and Kirby (1826: 311) as to the origin of the specimen can be explained by the fact that John Francillon and John Abbot (1752–1840) ‘of Georgia’ were known to each other, and Francillon acted as Abbott’s natural history agent. It is possible that Francillon mislabelled the specimen(s) or Kirby himself made the assumption as to the origin “Georgia, Amer.?”, (which we now know to be incorrect), possibly under the impression that Abbot had sent *Onthophagus* material to Francillon. Unfortunately, locality labels on specimens were infrequent during this early period in collections. The presumed male specimen mentioned in Kirby (1826: 311) has no labelling other than “cervicornis” in Kirby’s own distinctive hand (DJM confirmed by comparison with known Kirby handwritten labels).

**Figures 1–4.**
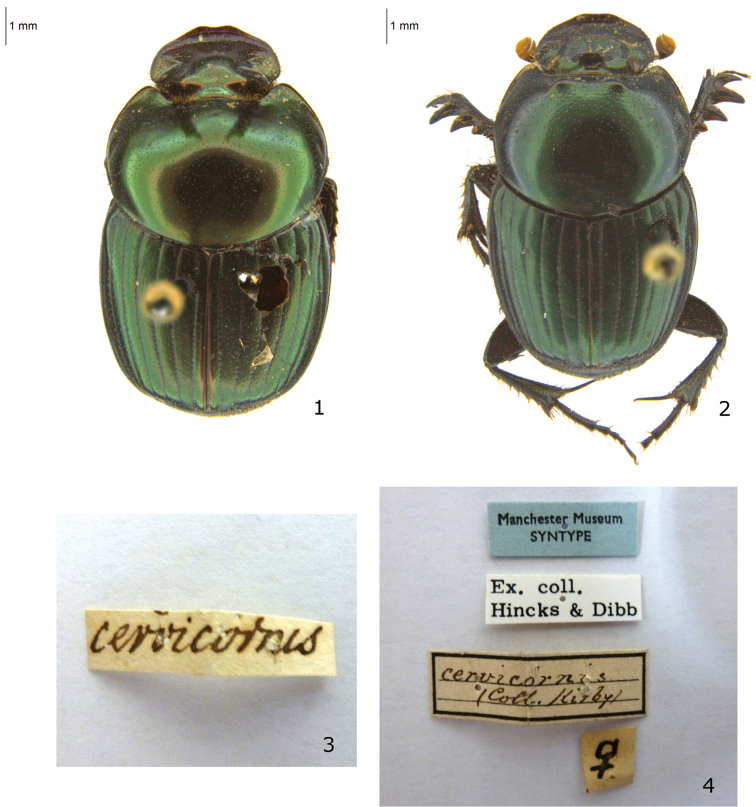
Syntypes of *Onthophagus cervicornis* Kirby, 1825. **1** Dorsal habitus of the male **2** Dorsal habitus of the female **3** Labels of the male (Kirby handwriting) **4** Labels of the female.

The collection of William Kirby (1759–1850) was presented to the Entomological Society of London during 1835, but was later (1858, 1863) sold at Stevens auction in lots (Chalmers-Hunt 1976: 102, Neave 1933: 71). Kirby specimens are now dispersed amongst a number of Museum Collections, with the bulk being housed in the Natural History Museum, London, and to a lesser extent the Oxford University Museum of Natural History. It is unclear how the Kirby material arrived at Manchester Museum. The female syntype of *Onthophagus cervicornis* is labelled with a modern type faced label “ex. coll. Hinks and Dibb” (Fig. 4), as is one other specimen. However, the majority of the *Onthophagus* specimens recognisable as Kirby’s (9 in total) do not possess similar labelling, suggesting that the later addition of these ‘accession’ labels may be mistaken. The female syntype of *Onthophagus cervicornis* (Fig. 2) was recognised as such by curators at the Manchester Museum and labelled as a syntype (Fig. 4). However, the male syntype (Fig. 1) remained unrecognised until now.

Harold (1869: 1030) appears to be the first author to consider *Onthophagus cervicornis* to be included under *Onthophagus janus* (Panzer, 1794), as he lists the first as a variety of the later. It was later followed by Horn (1875: 139) and others (e.g. Boucomont and Gillet 1927: 206, Howden and Cartwright 1963, Smith 2003: 30, Krajčik 2006: 134, Pulido-Herrera and Zunino 2007: 116), who considered both *Onthophagus cervicornis* and *Onthophagus janus* as synonyms under *Onthophagus striatulus* (Palisot de Beauvois, 1809). In their revision of the North American *Onthophagus*, the original description provided by Kirby led Howden and Cartwright (1963: 41) to consider that *Onthophagus cervicornis* was possibly African in origin, due to the branched horns of the male; nonetheless the authors retained the synonymy of *Onthophagus cervicornis*, as well as *Onthophagus janus* under *Onthophagus striatulus striatulus* (Palisot de Beauvois, 1809). In the most recent catalogue of new world *Onthophagus*, Pulido-Herrera and Zunino (2007: 116) maintain the synonymic placement of Howden and Cartwright (1963: 41).

## Material and methods

The morphological study was carried out using syntypes of *Onthophagus cervicornis* housed in the entomological collection of the Manchester Museum (MMUE, Dmitri Logunov) and currently on loan to one of us (DJM), as well as syntypes of *Onthophagus dama* (Fabricius coll.), formally ownership of the Zoological Museum of the University of Kiel, Germany, and permanently on loan to the Natural History Museum of Denmark (ZMUC, Alexey Y. Solodovnikov). Specimens were analysed and photographed with a stereomicroscope Leica M165 and a Leica DFC 490 digital camera attached. Pictures were firstly mounted with Helicon Focus 5.1 (Helicon Soft Ltd.) and then enhanced with GIMP 2.8 (www.gimp.org).

## Results and discussions

On examining syntypes of *Onthophagus cervicornis* the authors recognised that they correspond to a species widely known in collection as *Onthophagus dama* (Fabricius, 1798), a widespread and abundant species distributed across the Indian subcontinent.

### Onthophagus dama

Taxon classificationAnimaliaColeopteraScarabaeidae

(Fabricius, 1798)

Fig. 1–4

Copris dama Fabricius 1798: 32
Scarabaeus aeneus Olivier 1789: 131
Onthophagus dama (Fabricius 1798) Arrow 1931: 280
Onthophagus zubaci Balthasar 1932: 151
Onthophagus (Onthophagus) dama (Fabricius 1798) Balthasar 1963: 325
Onthophagus cervicornis Kirby 1825: 565, syn. n.

#### Remarks.

As no major nomenclatural concern can affect the current taxonomic status of *Onthophagus cervicornis*, we decided to maintain the syntypic status (Art. 73.2.1) for the examined specimens (ICZN 1999: 74.7.3). Instead, in order to maintain the nomenclature stability for *Onthophagus dama*, as well as the correct identification of further specimens, a male lectotype is here designated by choosing a name-bearing type specimen.

#### Geographical distribution.

Nepal, Bhutan, Sri Lanka and India: Chhattisgarh, Haryana, Himachal Pradesh, Karnataka, Madhya Pradesh, Maharashtra, Odisha, Tamil Nadu, Uttar Pradesh, Uttarakhand (Chandra and Gupta 2013: 4665), and Sikkim (Balthasar 1963: 326).

#### Type material examined.

Syntypes (1♂ + 1♀): 1♂, dry pinned. Original label: “cervicornis” [cream label, W. Kirby black handwritten]; “♂ syntype Onthophagus cervicornis Kirby 1825: 565 = Onthophagus dama (F.) Rossini & Mann, 2014” [printed] (MM). 1♀, dry pinned. Original labels: “♀” [cream label, black handwritten]/ cervicornis coll. Kirby [cream label with black border, black handwritten] / “Ex. Coll. Hincks & Dibb” [white label, black written printed] / “Manchester Museum, SYNTYPE” [blue label, black written printed] “♀ syntype Onthophagus cervicornis Kirby 1825: 565 = Onthophagus dama (F.) Rossini & Mann, 2014” [printed] (MM).

#### Ecology.

coprophagus, mostly attracted by cattle and human excrements, tunneler, active in any seasons and diurnal (Venugopal et al. 2012). Very widespread and abundant in tropical dry forest and agricultural habitats.

## Supplementary Material

XML Treatment for Onthophagus dama
